# The protective role of MLCP-mediated ERM dephosphorylation in endotoxin-induced lung injury *in vitro* and *in vivo*

**DOI:** 10.1038/srep39018

**Published:** 2016-12-15

**Authors:** Anita Kovacs-Kasa, Boris A. Gorshkov, Kyung-Mi Kim, Sanjiv Kumar, Stephen M. Black, David J. Fulton, Christiana Dimitropoulou, John D. Catravas, Alexander D. Verin

**Affiliations:** 1 Vascular Biology Center, Medical College of Georgia at Augusta University, Augusta, Georgia; 2 Center for Lung Vascular Pathobiology, University of Arizona, Phoenix, Arizona; 3 Frank Reidy Center for Bioelectrics, Old Dominion University, Norfolk, Virginia

## Abstract

The goal of this study was to investigate the role of MLC phosphatase (MLCP) in a LPS model of acute lung injury (ALI). We demonstrate that ectopic expression of a constitutively-active (C/A) MLCP regulatory subunit (MYPT1) attenuates the ability of LPS to increase endothelial (EC) permeability. Down-regulation of MYPT1 exacerbates LPS-induced expression of ICAM1 suggesting an anti-inflammatory role of MLCP. To determine whether MLCP contributes to LPS-induced ALI *in vivo,* we utilized a nanoparticle DNA delivery method to specifically target lung EC. Expression of a C/A MYPT1 reduced LPS-induced lung inflammation and vascular permeability. Further, increased expression of the CS1β (MLCP catalytic subunit) also reduced LPS-induced lung inflammation, whereas the inactive CS1β mutant increased vascular leak. We next examined the role of the cytoskeletal targets of MLCP, the ERM proteins (Ezrin/Radixin/Moesin), in mediating barrier dysfunction. LPS-induced increase in EC permeability was accompanied by PKC-mediated increase in ERM phosphorylation, which was more prominent in CS1β-depleted cells. Depletion of Moesin and Ezrin, but not Radixin attenuated LPS-induced increases in permeability. Further, delivery of a Moesin phospho-null mutant into murine lung endothelium attenuated LPS-induced lung inflammation and vascular leak suggesting that MLCP opposes LPS-induced ALI by mediating the dephosphorylation of Moesin and Ezrin.

Acute lung injury (ALI) is a critical hypoxemic respiratory failure characterized by vascular leak, alveolar capillary membrane damage, severe inflammation and pulmonary edema secondary to endothelial barrier dysfunction^1^. ALI has a high incidence among the US population and mortality rates remain excessive^2^. During ALI, bacterial sepsis is a major cause of mortality and bacterial toxins like LPS (lipopolysaccharide) and the endotoxins of Gram-negative bacteria^3^ contribute to inflammation and endothelial barrier dysfunction. LPS binds and activates TLR4 (Toll-like receptor 4) and promotes the activation of NFκB and MAPK leading to the increased synthesis and release of inflammatory cytokines or type I interferon. LPS also stimulates PKC^4^ signaling and activates the small GTPase Rho^5^ resulting in actin cytoskeleton remodeling and the loss of endothelial barrier integrity.

Endothelial cells (EC) form a semi-permeable barrier between the circulating blood and the surrounding tissues. Endothelial cells regulate the traffic of cells and fluids to the extravascular space through changes in permeability. Loss of EC barrier function, a prominent feature of ALI, is tightly linked to agonist-induced cytoskeletal reorganization which disrupts cell-cell contacts, and promotes the formation of paracellular gaps^6^,^7^,^8^.

One of the major factors underlying increases in EC permeability is the actomyosin-driven contraction of EC. EC contraction is initiated by the reversible phosphorylation of the 20 kDa myosin regulatory light chain (MLC) at Ser19 and Thr18 amino acid residues, which is tightly linked to F-actin filament reorganization^9^. MLC phosphorylation is regulated by the activities of MLC kinases (MLCK and Rho-kinase) and MLC phosphatase (MLCP)^10^,^11^. The role of MLCK and Rho-kinase in the EC barrier dysfunction *in vitro* and *in vivo* is well documented^12^,^13^. However, the importance of MLCP which counteracts these mechanisms via dephosphorylation, in the regulation of the EC barrier remains largely unexplored. MLCP is a type-1 protein phosphatase (PP1), composed of the PP1 catalytic subunit (CS1β formerly CS1δ) and two non-catalytic subunits, the 20 kDa small regulatory subunit (M20) and a 110–130 kDa regulatory subunit, called myosin phosphatase targeting subunit 1 (MYPT1)^14^,^15^. We have previously shown that MLCP-mediated signaling plays an important role in EC barrier strengthening induced by extracellular purines *in vitro*^16^,^17^,^18^. However, its role in the regulation of lung vascular permeability in an LPS model of ALI is unknown.

We and other groups have previously reported that Ezrin/Radixin/Moesin (ERM) proteins are substrates for MLCP^18^,^19^,^20^. ERM proteins are members of the protein 4.1 superfamily and are characterized by a plasma membrane associated FERM domain at the N-terminus and an F-actin binding C-terminal ERM association domain^21^. ERMs function as linkers between the plasma membrane and the actin cytoskeleton, and are involved in many crucial cellular functions such as cell adhesion, membrane ruffling and microvilli formation^21^,^22^. ERM proteins can be phosphorylated on several Ser/Thr and Tyr residues^23^,^24^ and the most-studied sites are the conserved Thr residues at the C terminal (Thr558 in Moesin, Thr576 in Ezrin and Thr564 in Radixin). The phosphorylation of these Thr residues on the C terminus activates ERMs by reducing the affinity between the transmembrane N-terminus and the ERM association domain. Activated ERMs are able to bind their target proteins (F-actin or protein kinases)^25^.

While we and others have recently shown that the phosphorylation/dephosphorylation of ERMs is involved in EC cytoskeletal regulation in various permeability models *in vitro*^20^,^26^ their role in the regulation of LPS-induced EC permeability has not been reported.

In this study our goal was to define the role of MLCP and its cytoskeletal targets, the ERM proteins, in the LPS model of lung injury *in vitro* and *in vivo*. For the first time, we demonstrate that MLCP is involved in vascular endothelial barrier protection against LPS-induced EC barrier disruption *in vitro* and *in vivo* in a murine model of ALI. We show that LPS increases ERM and MLC phosphorylation via the activation of different pathways. Moesin and Ezrin, but not Radixin, are directly involved in LPS-induced EC barrier disruption *in vitro.* Further, introduction of phospho-null Moesin mutant into the mouse lung significantly attenuates LPS-induced lung inflammation and vascular leak suggesting that Moesin dephosphorylation may protect against lung injury in a LPS-mediated murine model of ALI *in vivo*.

## Results

### MLCP is involved in endothelial barrier protection against LPS-induced lung vascular injury *in vitro* and in murine model of ALI

**Figure 1:**
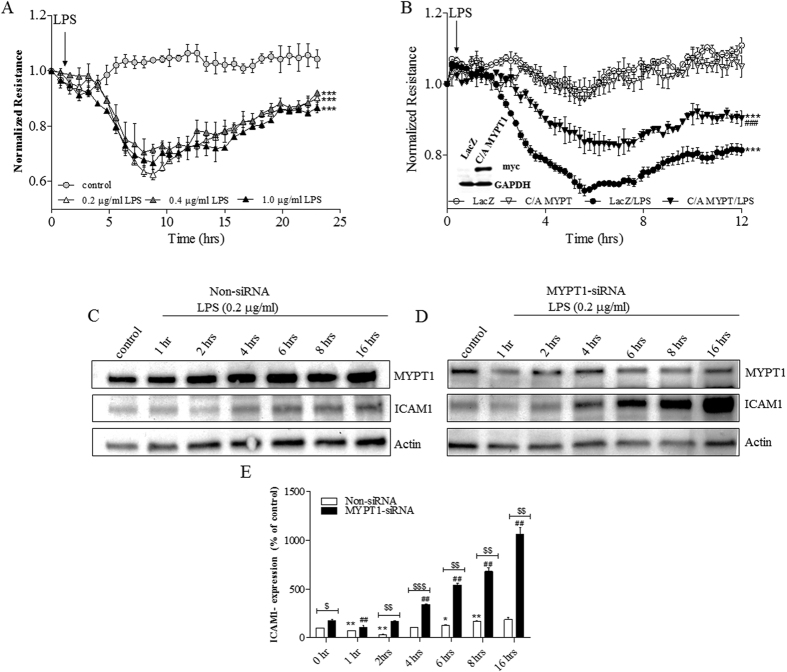
(**A**) Effect of different concentrations of LPS on TER in HPAEC. HPAEC were plated on gold microelectrodes in ECIS arrays (8W10E+). Once a constant resistance was reached, the cells were treated with either vehicle (0.1% DMSO) or different concentrations of LPS of (0.2, 0.4 or 1 μg/ml) at indicated time, and TER was monitored for 24 hours. (**B**) Effect of MLCP activation on LPS-induced permeability increase. HPAEC were transduced with LacZ (control) or C/A MYPT1 adenovirus (20 MOI), and 24 hours later cells were plated on ECIS arrays (8W10E+). After reaching a constant resistance, cells were treated with either vehicle (0.1% DMSO) or LPS (0.2 μg/ml) at indicated time and TER was monitored for 12 hours. Normalized resistance was calculated and plotted as a function of time. The overexpression of C/A MYPT1 was detected by Western blot (inset). ***P < 0.0001 vs. control; ^###^P < 0.0001 vs. C/A MYPT1/LPS. (**C–E**) Effect of MLCP down-regulation on LPS-induced ICAM1 expression. (**C,D**) Representative immunoblots of MYPT1 and ICAM1 showing ICAM1 and MYPT1 expression. HPAEC were transfected with non-targeting siRNA (**C**) or MYPT1 specific siRNA (**D**). After 72 hours incubation, cells were treated with LPS (0.2 μg/ml) for different time periods (0, 1, 2, 4, 6, 8, 16 hours) and western blot analysis was performed. (**E**) Bar graph showing changes in protein levels of ICAM1 quantified with scanning densitometry. Results are expressed as Mean ± SEM (n = 5 experiments). *P < 0.05, **P < 0.01 vs. non-siRNA transfected control; ^##^P < 0.01, vs. MYPT1-siRNA transfected control; ^$$^P < 0.01, ^$$$^P < 0.0001 non-siRNA/LPS vs. MYPT1-siRNA/LPS.

Our group has previously shown that MLCP has a critical role in EC barrier strengthening induced by extracellular purines^16^,^17^,^18^ and in protection against thrombin-induced EC barrier compromise *in vitro*^18^. To examine the role of MLCP in EC barrier compromise in a cell culture model we first analyzed LPS-induced permeability changes to identify the effective dose of LPS in human pulmonary artery endothelial cells (HPAEC). We show that LPS concentrations in the range of 0.2–1 μg/ml induced comparable decreases in TER with maximal effect at 6–8 hours of treatment (Fig. 1A). Consequently, we used the lowest concentration (0.2 μg/ml) in all subsequent experiments. To determine whether MLCP activity has a protective role in the LPS model of EC barrier compromise, we performed ECIS experiments with EC overexpressing the C/A truncated MYPT1 (C/A MYPT1 1–300). HPAEC were transduced with LacZ (control) or with C/A MYPT1-myc adenovirus. 24 hours after infection, HPAEC were treated with LPS or vehicle-control, and TER was monitored for 12 hours. We found that the overexpression of C/A MYPT1 attenuates LPS-mediated decrease in HPAEC barrier function (Fig. 1B). *These results are consistent with our previously published observations indicating that depletion of MYPT1 significantly attenuated HPAEC barrier enhancement induced by extracellular purines (ref.* 17, 18). To further define the role of MLCP in LPS-induced ALI *in vitro,* we examined the effect of MYPT1 depletion on ICAM1 expression in LPS-treated HPAEC (Fig. 1C–E). Our results demonstrate that LPS increases ICAM1 expression levels after 4 hours in a time-dependent manner in non-targeting siRNA-transfected control cells (Fig. 1C). MYPT1 depletion exacerbated the effect of LPS (Fig. 1D) strongly supporting the direct anti-inflammatory role of MLCP in this LPS model of endothelial injury.

**Figure 2:**
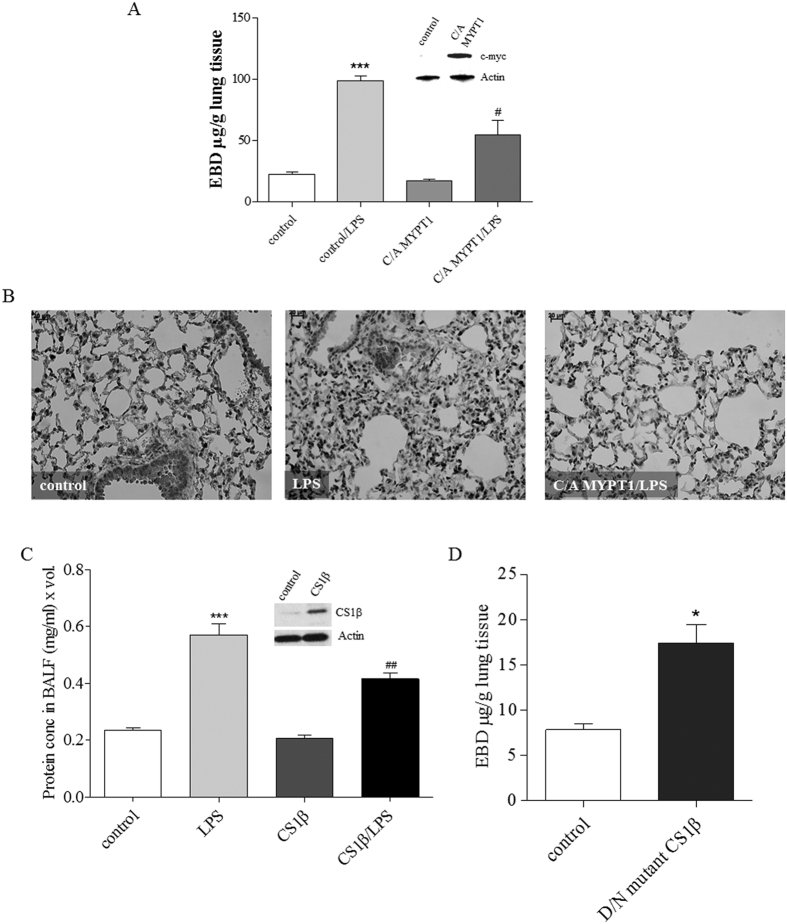
C/A MYPT1 expression attenuates LPS-induced lung inflammation *in vivo*. (**A**) A plasmid (40 μg) encoding C/A MYPT1 (1–300) fragment or empty pcDNA 3.1 (40 μg) was complexed with JetPEI and injected via a tail vein. LPS (1 mg/kg) was administered intratracheally (i.t.) 72 h later, then capillary leak was evaluated using Evans blue dye. Bar graph shows EBD (μg/g) in lung tissue, ***P < 0.0001 pcDNA 3.1 vs. pcDNA 3.1/LPS; ^#^P > 0.05 control LPS vs. C/A MYPT1/LPS. Western blot shows the expression level of C/A MYPT1 in mouse lungs injected with pcDNA3.1 control plasmid or with C/A MYPT expression plasmid using anti-c-myc antibody and actin as a loading control. (**B**) MPO staining of control, LPS, C/A MYPT1/LPS mouse lung samples. Plasmid encoding wild type (**C**) or mutant (**D**) CS1β was complexed with jetPEI then injected i.v., and 72 hours later LPS (1 mg/kg) was administered i.t. Protein concentration in BALF (**C**) and EBD (**D**) were evaluated. Western blot shows the expression level of CS1β in mouse lungs. The samples from (**C**) and (**D**) derive from the same experiment and that gels/blots were processed in parallel. Results are expressed as Mean ± SEM (n = 5 experiments). *P < 0.05 control vs. mutant CS1β, ***P < 0.0001 control vs. LPS; ^#^P < 0.05, ^##^P < 0.01, LPS vs. CS1β/LPS.

To evaluate whether MLCP activity has a barrier-protective and anti-inflammatory role in LPS-induced vascular barrier injury *in vivo,* we utilized a LPS murine model of ALI in conjunction with a lung-targeted gene delivery method which we have recently developed^27^. In this approach, commercial (Polyplus) cationic polymer jetPEI (polyethyleneimine) transfection reagent forms a complex with the DNA of interest that specifically targets the lungs with preferential localization to the endothelium^27^. Utilizing this approach, plasmid encoding C/A MYPT1 1–300 or vector control (empty pcDNA 3.1) complexed with JetPEI was introduced intravenously (i.v.). Three days (72 hrs) later, LPS or vehicle was instilled intratracheally (i.t.). Capillary leak was estimated using Evans Blue Dye-conjugated albumin, and lung inflammation was evaluated by MPO staining as described in Materials and Methods. The efficiency of the *in vivo* gene delivery was confirmed by immunoblotting of lung homogenates with c-myc-specific antibody. Results in Fig. 2 demonstrate that overexpression of C/A MYPT1 significantly attenuates LPS-mediated lung vascular permeability increase (Fig. 2A). Furthermore, ectopic expression of C/A MYPT1 dramatically decreased lung inflammation caused by LPS (Fig. 2B). Therefore, our results indicate that MLCP plays an anti-inflammatory and barrier-protective role against LPS-induced ALI in mice.

To further confirm our findings, we introduced expression plasmids containing either wild type CS1β (C/A) or the dominant negative (D/N) mutant CS1β (MLCP catalytic subunit) into mouse lungs using the JetPEI-assisted delivery approach. Our results demonstrate that wild type CS1β significantly reduces LPS-induced inflammatory processes in the mouse lung, as inferred from a reduction of protein content in BALF (Fig. 2C). Conversely, introduction of the D/N CS1β mutant significantly increases vascular leak (Fig. 2D). Collectively, our present results strongly implicate the involvement of MLCP activity in EC barrier strengthening *in vitro* and in a murine model of LPS-induced ALI.

### LPS-induced increases in MLC and ERM phosphorylation involves activation of different signaling pathways

We have previously shown that MLCP substrates, MLC and ERM proteins, are involved in EC barrier regulation in various endothelial permeability models^18^,^20^,^28^. To clarify the involvement of LPS in MLC and ERM phosphorylation and find a link between MLC and ERM phosphorylation level changes we analyzed the levels of di-phospho-MLC and phospho-ERM in HPAEC. ERM and MLC phosphorylation in LPS-challenged endothelial cells was analyzed by immunoblotting at discrete time points (0, 1, 2, 4, 6, 8 hours) utilizing an anti-di-phospho MLC antibody (Thr 18/Ser19) and a phospho-specific ERM antibody (phospho-Ezrin Thr567/Radixin Thr564/Moesin Thr558). Our data demonstrate that LPS induced a gradual increase in Thr phosphorylation of ERMs which correlated with LPS-induced permeability changes (Fig. 1A). In contrast, the increase in MLC phosphorylation (maximum at 2–4 hours) preceded the maximal LPS-mediated EC barrier compromise (Fig. 1A). This data suggest that, similar to the thrombin model^29^, the increase in MLC phosphorylation may be involved in the initiation of LPS-caused EC barrier compromise, whereas increase in ERM phosphorylation is important for the development of the maximal response. Therefore, LPS-induced increases in MLC and ERM phosphorylation require activation of different signaling pathways[Fig f3].

To confirm that ERM are substrates of MLCP in HPAEC, we depleted the MLCP catalytic subunit, CS1β, utilizing a siRNA approach. HPAEC were transfected with a specific-CS1β siRNA or a scrambled siRNA control, then treated with LPS for the indicated time periods, and the level of ERM phosphorylation was determined by immunoblotting with a phospho ERM antibody, as described in “Materials and Methods”. Our results show that depletion of CS1β significantly increased ERM phosphorylation in control and LPS-treated HPAEC, compared to the scrambled siRNA transfected cells (Fig. 4A–C).

To evaluate the kinases involved in LPS-induced MLC and ERM phosphorylation, we used pharmacological approach. We applied Rho-kinase inhibitor, Y-27632 (10 μΜ), PKC-inhibitor, Ro-31–7459 (10 μΜ) or DMSO-control (0.1%) one hour before adding LPS (0.2 μg/ml) for six hours. Inhibition of PKC, but not Rho-kinase, abolishes LPS-induced ERM phosphorylation in human EC. In contrast, Rho-kinase, but not PKC, is involved in LPS-induced MLC phosphorylation (Fig. 5A). Therefore, we identified PKC, but not Rho kinase as a major kinase involved in ERM phosphorylation in LPS-challenged EC. In contrast, the major kinase responsible for MLC phosphorylation is Rho kinase, as expected.

### The role of ERM proteins in LPS-induced EC barrier dysfunction

Our recently published results demonstrate that ERM proteins play important, but different roles in the thrombin-induced EC barrier dysfunction: Moesin, and to a lesser extent Ezrin promote barrier dysfunction, and Radixin opposes it^28^. However, we have also shown that individual ERM protein depletion significantly attenuates the EC permeability increase induced by the microtubule disruptor, 2-methoxyestradiol (2ME)^20^. Further, HPAEC monolayers overexpressing phospho-null ERM mutants, exhibit less attenuation of 2ME-induced hyper-permeability, in response to PKC inhibition, implicating the phosphorylation of all ERMs in EC barrier dysfunction^20^. Collectively, this data suggest that involvement of ERM proteins in EC barrier regulation is likely to be agonist-specific. To examine the functional role of individual ERM proteins in LPS-induced EC barrier dysfunction, TER was used to detect permeability changes in Moesin-, Ezrin- or Radixin-depleted HPAEC. Non-targeting siRNA transfected cells were used as controls. The efficiency of the depletion of each protein was confirmed by immunoblotting. Our data demonstrates that Moesin (Fig. 6A) and Ezrin (Fig. 6B) depletion significantly attenuated the effect of LPS on TER, while depletion of Radixin slightly decreased basal TER, but did not significantly alter the LPS response (Fig. 6C). Therefore, our data demonstrates that ERM proteins have different roles in LPS-induced EC barrier compromise and confirms that the involvement of these proteins in EC barrier regulation may be agonist-specific.

### The role of Moesin phosphorylation in LPS-induced vascular barrier dysfunction *in v*i*vo*

**Figure 3 f3:**
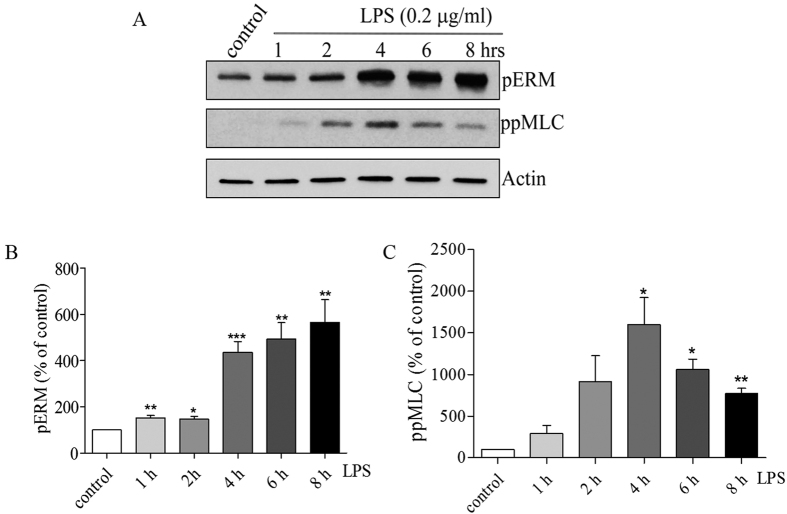
LPS induces phosphorylation of ERM and MLC in a time-dependent manner. (**A**) Western blot analysis of the phosphorylation level of ERM proteins and phospho-MLC in HPAEC. Actin served as a loading control. HPAEC were treated with LPS (0.2 μg/ml) for the indicated time periods. (**B/C**) Bar graph shows the changes in protein levels of phospho-ERM and phospho-MLC quantified with scanning densitometry. Results are expressed as Mean ± SEM (n = 4 experiments). *P < 0.05, **P < 0.01, ***P < 0.0001 vs. control.

**Figure 4:**
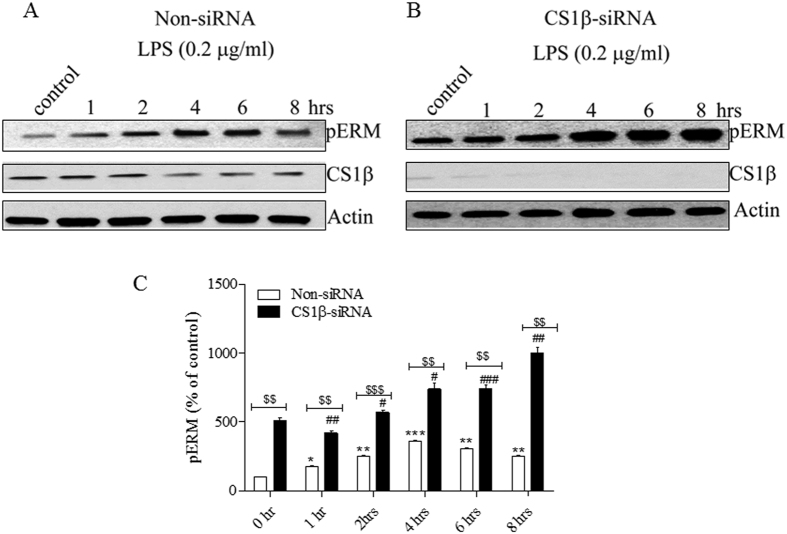
MLCP activity plays an important role in ERM dephosphorylation. (**A,B**) Representative immunoblots showing the levels of pERM and CS1β expression. HPAEC monolayers were transfected using non-targeting siRNA (**A**) or CS1β specific siRNA (**B**). After 72 hours, cells were treated with LPS (0.2 μg/ml) for different time periods and western blot analysis was performed using anti phospho-ERM and CS1β antibodies. The samples from (**A**) and (**B**) derive from the same experiment and that gels/blots were processed in parallel. (**C**) Bar graph shows the changes in phospho-ERM content quantified with scanning densitometry. Results are expressed as Mean ± SEM (n = 3). *P < 0.05, **P < 0.01, ***P < 0.0001 vs. non-siRNA transfected control; ^#^P < 0.05, ^##^P < 0.01, ^###^P < 0.0001 vs. CS1β -siRNA transfected control; ^$$$^P < 0.0001 non-siRNA/LPS vs. CS1β -siRNA/LPS.

**Figure 5:**
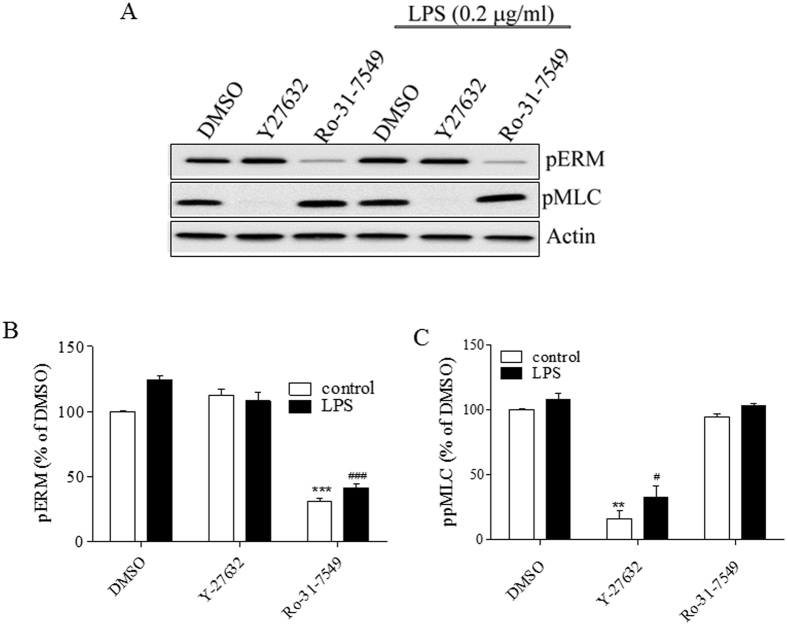
LPS-induced phosphorylation of ERM and MLC involves different signaling. HPAEC were pretreated with ether a Rho-kinase (Y-27632, 10 μΜ), or a pan PKC (Ro-31-7459, 10 μΜ) inhibitor, or with DMSO (0.1%), one hour before adding LPS (0.2 μg/ml). At designated time, cells were harvested and analyzed by immunoblotting. (**A**) Representative immunoblots show the phosphorylation levels of phospho-ERM and phospho-MLC. (**B/C**) Bar graphs show the changes in the levels of phospho-ERM and phospho MLC quantified with scanning densitometry. Results are expressed as Mean ± SEM (n = 5 experiments). **P < 0.01, vs. DMSO control; ^#^P < 0.05, ^###^P < 0.0001 vs. DMSO control/LPS.

**Figure 6:**
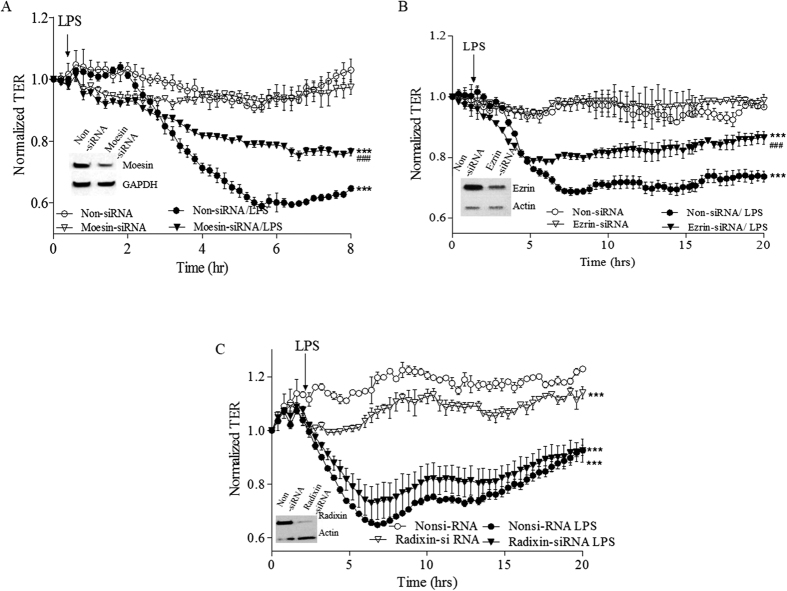
Moesin and Ezrin, but not Radixin depletion significantly attenuates effect of LPS on TER. HPAEC was plated on small gold electrodes in ECIS chamber and were transfected with siRNAs (50 nM) to deplete (**A**) Moesin, (**B**) Ezrin, (**C**) Radixin, and using SiPort-based delivery. Three days after transfection, TER was measured. After stabilization for 1 hour, cells were challenged with LPS (0.2 μg/ml), and the change in TER was recorded for 8–20 hours. Non-targeting RNA (Non-siRNA) was used as a control. Results are expressed as Mean ± SEM, n = 3 experiments. Normalized resistance was calculated and plotted as a function of time. **Insets**. Representative immunoblots showing the level of individual ERM protein depletion. ***P < 0.0001 vs. control; ^###^P < 0.0001 vs. Moesin or Ezrin or Radixin- siRNA/LPS.

**Figure 7:**
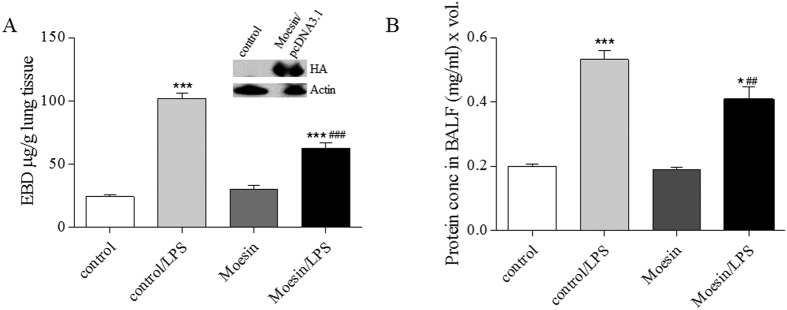
Moesin phosphorylation plays an essential role in LPS-induced lung injury *in vivo*. PcDNA3.1 plasmids (40 μg) encoding empty vector (control) or unphosphorylatable Moesin mutant were complexed with JetPEI transfection reagent. Three days later, LPS (1 mg/kg) was injected i.t. (**A**) Bar graph shows Evans Blue dye extravasation, reflecting capillary leak. Western blot of lung homogenates stained for HA-tagged Moesin-mutant. (**B**) Bar graph shows protein levels in BALF (an index of lung inflammation and capillary leak). *P < 0.05, ***P < 0.0001 vs. control, ^##^P < 0.01, ^###^P < 0.0001 vs. control/LPS.

We have previously shown that Moesin is the predominant ERM protein in HPAEC^28^. It is involved in EC barrier compromise in several permeability models including LPS-caused EC barrier dysfunction *in vitro*^20^,^28^ (Fig. 3A). Moesin is activated by phosphorylation at Thr558^25^. To assess the role of Moesin phosphorylation in vascular barrier regulation *in vivo* we generated a phospho-null Moesin mutant (Thr558 to Ala^18^) and utilized an *in vivo* LPS murine model of ALI in conjunction with jetPEI delivery method, as we have recently described^27^. Mammalian expression plasmids coding either phospho-null Moesin mutant in pcDNA 3.1 or a control empty pcDNA 3.1 plasmid were injected into mice via a tail vein^27^. The overexpression of phospho-null Moesin mutant significantly decreased lung permeability inferred from EBD extravasation (Fig. 7A) and also decreased the protein level in BALF (Fig. 7B) from mouse lungs treated with LPS, indicative of lung inflammation. This data demonstrated for the first time that Moesin phosphorylation is involved in LPS-induced ALI *in vivo*, therefore supporting barrier-protective role of MLCP (which dephosphorylate Moesin) *in vitro* and *in vivo*.

## Discussion

Endotoxin lipopolysaccharide (LPS), a constituent of the cell wall of Gram-negative bacteria, is a potent pro-inflammatory agent that directly disrupts EC barrier function in both macro- and microvascular EC^5^,^30^ LPS-mediated murine lung injury, is a disease model that shares many characteristics of sepsis-induced ALI/ARDS in humans. Specifically, the injury elicited is characterized by neutrophil infiltration into the lung in association with increased pro-inflammatory mediators including nuclear factor (NF)-κB which leads to the up-regulation of pro-inflammatory cytokines such as tumor necrosis factor (TNF)-α, IL-6, IL-8, the up-regulation of ROS and RNS generators (NADPH oxidase, iNOS), increased formation of protein-rich alveolar fluid (edema), structural damage to the alveolo-capillary membrane and compromised lung function^31^,^32^.

It has been well established that edemagenic agents like thrombin or histamine induce cytoskeletal rearrangement via MLCK, PKC and activation of RhoA leading to the formation of paracellular gaps and the loss of EC barrier integrity^7^,^33^,^34^. The precise mechanisms by which LPS-induced signaling leads to EC barrier disruption are not yet fully clarified. However, it is clear that LPS acts through the activation of cell surface TLR4 which leads to reorganization of the actin cytoskeleton and increased EC permeability (reviewed in ref. 8). Similar to thrombin, LPS promotes cytoskeletal changes that are dependent, at least in part, on the activation of PKC and Rho signaling pathways^35^,^36^,^37^. Rho/Rho-kinase activation leads to increased MLC phosphorylation and EC contraction. Rho-kinase phosphorylates MLC directly on Ser19 and (or) indirectly via increased phosphorylation of the inhibitory site of MYPT1 leading to MLCP inhibition and a subsequent increase in MLC phosphorylation at Thr18/Ser19^8^,^38^. PKC activation can also lead to increased MLC phosphorylation via activation of the MLCP inhibitor, CPI-17 (PKC potentiated inhibitory protein of 17 kDa) or activation of the Rho pathway via PKC-mediated inhibition of the Rho inhibitor, GDI-1^34^,^39^,^40^. However, the latter signaling pathways have not been defined in a LPS permeability model. Other putative cytoskeletal targets of PKC include the actin binding proteins caldesmon and the ERM proteins (reviewed in ref. 8), however, their role in LPS-induced lung injury is also unknown.

Pathways that can protect the endothelial barrier against LPS-induced lung injury are far less understood. Our recently published data have demonstrated barrier-protective effects of HSP-90 inhibitors, DDAH II (dimethylaminohydrolase II) overexpression, microtubule stabilization, extracellular β-NAD (β-nicotinamide adenine dinucleotide) and extracellular purines, ATP and adenosine^27^,^35^,^41^,^42^,^43^,^44^. We have previously shown that purine-induced EC barrier strengthening is tightly linked to activation of MLCP and MLCP is involved EC barrier protection against thrombin-induced EC barrier compromise *in vitro*^16^,^17^,^18^. However, the role of MLCP in LPS-induced lung injury *in vitro* and *in vivo* has not been described and was the aim of the current study.

We demonstrated that the ectopic expression of a C/A MLCP regulatory subunit (truncated MYPT1, 1–300) significantly attenuates the LPS-induced decreases in TER reflecting EC barrier protection. In contrast, down-regulation of MYPT1 leads to exacerbation of LPS-induced expression of ICAM-1 suggestive of a direct anti-inflammatory function of MLCP. To further define the role of MLCP in LPS-induced ALI *in vivo,* we utilized a nanoparticle DNA delivery method which we recently developed^27^ to specifically target lung EC. Using this method, we demonstrated that ectopic expression of C/A MYPT1 reduces lung inflammation (MPO staining) and attenuates LPS-induced vascular permeability (Evans Blue Dye albumin (EBA) extravasation into the lung). Consistent with these results, delivery of plasmids encoding the wild type CS1β (MLCP catalytic subunit) significantly reduced LPS-induced lung inflammation as determined by the reduction of protein content in bronchoalveolar lavage fluid (BALF). Conversely, the *in vivo* transfection of a catalytically inactive (D/N) CS1β mutant increases vascular leak. Collectively, these data convincingly demonstrate for the first time that MLCP directly provides EC barrier protection against LPS-induced EC barrier disruption *in vitro* and LPS-induced vascular leak *in vivo.* In addition, they support an anti-inflammatory role of MLCP *in vitro* and *in vivo*.

MLC is the classical substrate for MLCP. MLCP-mediated MLC dephosphorylation is primarily regulated by MYPT1 binding to myosin^14^,^15^,^45^. However, more recent studies indicate that additional cytoskeletal proteins may be targets for MLCP and suggest that MLCP may have a broader multidirectional involvement in the control of EC cytoskeleton and permeability^12^,^46^,^47^.

ERM (Ezrin/Radixin/Moesin) proteins are unconventional MLCP cytoskeletal substrates^19^,^48^ ERM proteins act both as linkers between the actin cytoskeleton and plasma membrane, and as signal transducers in responses involving cytoskeletal remodeling^49^,^50^,^51^. In the unphosphorylated form, ERM proteins exist in auto-inhibited conformation. Phosphorylation induces and stabilizes the unfolded active conformation of ERMs, allowing them to simultaneously bind to membrane and actin filaments^51^,^52^. MYPT1 can bind to ERM proteins and facilitates MLCP-dependent ERM dephosphorylation^19^. Importantly, we and others have shown that ERM proteins are involved in EC barrier regulation^20^,^26^,^28^,^53^, however, their role in LPS-induced EC barrier compromise was unknown. Our data demonstrate that LPS-induced ERM phosphorylation occurs in parallel with the reduction in TER. In contrast, LPS-induced MLC phosphorylation is transient with a nadir at 2–4 hrs, which precedes maximal changes in permeability. These data suggest that LPS-induced MLC phosphorylation is involved in the initiation of the contractile response and mediates permeability increases, whereas ERM phosphorylation may be important for the magnitude and/or duration of permeability increases. Our data also demonstrate that LPS-induced increases in ERM phosphorylation are more prominent in CS1β-depleted cells and confirm that ERMs are cytoskeletal targets for MLCP in EC.

ERM proteins are phosphorylated at conserved Thr residues on the C-terminus (Thr 558 for Moesin, Thr567 for Ezrin and Thr564 for Radixin). The established literature indicate that multiple kinases are involved in the phosphorylation of ERMs in different cell types and in response to various stimuli. Initially Rho-kinase and PKC were the only kinases reported to phosphorylate ERM^54^,^55^,^56^. Lately, several other kinases have been identified that phosphorylate ERMs in different cell types^57^. Importantly, studies on lymphocytes demonstrate that the kinase(s) responsible for ERM phosphorylation may be cell-specific^57^.

According to a recent publications, ERM phosphorylation in EC is dependent on PKC, p38, and Rho-kinase either alone or in combination^26^,^53^. We have recently shown that thrombin-induced EC barrier dysfunction is accompanied by PKC-mediated ERM phosphorylation^28^. In addition, we have demonstrated that the microtubule disruptor, 2-methoxyestradiol (2ME), increases ERM phosphorylation in a p38/PKC-dependent fashion. The activation of p38 appears to occur upstream from the activation of PKC^20^. In contrast, TNFα-induced increases in ERM phosphorylation and EC barrier dysfunction likely involves parallel activation of p38 and PKC-mediated signaling^53^. Our data demonstrate that LPS-induced ERM phosphorylation is PKC, but not Rho-kinase dependent. At the same time we have demonstrated that LPS-induced MLC phosphorylation is Rho-kinase, but not PKC-dependent. These data indicate that LPS-induced ERM and MLC phosphorylation require the activation of different signaling pathways and support the hypothesis that MLC and ERM phosphorylation may be involved in different phases of LPS-induced EC permeability responses.

We and others have recently demonstrated that despite structural similarity among the ERM proteins, their individual roles in EC barrier function may vary and the degree of involvement may be agonist-specific^26^,^28^. These observations highlight the critical need to examine the role of individual ERM proteins in LPS-induced EC barrier disruption. Using short interfering RNA to specifically deplete Ezrin, Moesin and Radixin in EC, we demonstrated that depletion of Moesin and Ezrin significantly attenuates LPS-induced decrease in TER. In contrast, depletion of Radixin, but not Moesin and Ezrin modestly decreases baseline TER of unstimulated EC monolayers and has no significant effect on LPS-induced increases in permeability. The origin of the functional differences between highly homologous ERM proteins are not entirely clear, but most likely arises from differences in the interactions with partnering regulatory proteins. For example, Moesin and Ezrin differentially bind to L-selectin in lymphocytes^58^; Radixin, but not Ezrin or Moesin bind to the integral membrane hyaluronan receptor, layilin^59^. Further, we have recently shown that ERM proteins have a different binding affinity toward the subunits of MLCP, CS1β and MYPT1. Moesin and to a lesser extent Ezrin, but not Radixin can be detected in CS1β immunoprecipitates. In contrast, Radixin, but not Ezrin or Moesin co-immunoprecipitates with MYPT1 in COS7 cells^18^. These differences in the binding capacities of ERM proteins for MLCP subunits may contribute to the different functional roles of ERM proteins in EC barrier regulation. However, the exact role of differential ERM-MLCP binding both at baseline and following LPS-stimulation remains to be determined.

Using phosphorylation-deficient ERM mutants that were ectopically expressed in EC, we have recently demonstrated a direct role of ERM phosphorylation in the loss of EC barrier integrity induced by the microtubule inhibitor, 2ME, *in vitro*^20^. To evaluate the role of ERM phosphorylation in LPS-induced vascular barrier dysfunction *in vivo* we delivered an expression plasmid encoding a phospho-null Moesin mutant into murine lung using a nanoparticle based DNA delivery method^27^. Our data demonstrate that ectopic expression of the phosphorylation-deficient Moesin significantly attenuates LPS-induced lung inflammation and vascular leak supporting a regulatory role of changes in Moesin phosphorylation in mediating changes in vascular barrier permeability in response to LPS. To the best of our knowledge, this is the first report directly showing a role of Moesin phosphorylation/dephosphorylation in the regulation of lung vascular barrier function *in vivo.*

Collectively, our data demonstrated that MLCP opposes LPS-induced lung injury *in vitro* and *in vivo*, at least in part, via dephosphorylation of its cytoskeletal targets, the ERM proteins.

## Materials and Methods

### Materials

All chemicals were purchased from Sigma-Aldrich (St. Louis), unless otherwise indicated. LPS is *E. coli* serotype 0127:B8, endotoxin level 3,000,000 endotoxin units/mg. Antibodies were from Cell Signaling Technology, Inc. (Danvers, MA): phospho-Ezrin (Thr567)/Radixin (Thr564)/Moesin (Thr558) rabbit monoclonal antibody, MYPT1 rabbit monoclonal antibody, GAPDH rabbit monoclonal antibody, p-Myosin Light Chain 2 (Thr18/Ser19) rabbit polyclonal antibody, Ezrin, Radixin, Moesin rabbit monoclonal antibodies. Jet-PEI reagent was obtained from Polyplus-transfection Inc. (New York, NY). Myeloperoxidase (MPO) assay kit was purchased from Dako North America, Inc. (Carpinteria, CA). The Rho kinase inhibitor Y-27632 and the PKC inhibitor Ro-31-7549 were purchased from Calbiochem (San Diego, CA). ECIS arrays (8W10E+) were from Applied BioPhysics (Troy, NY). Transfection plasmids were generated in our laboratory as we have previously described^18^. The phospho-null Moesin mutant was generated by Stratagene (San Diego, CA) using our wild type construct as a template. Plasmid containing C/A MYPT1 (amino acids 1–300), which was generously provided by Dr. M. Eto (Thomas Jefferson University, Philadelphia, PA), was used as a template for subcloning of C/A MYPT1 (1–300) into an adenoviral vector. Construction of adenovirus containing C/A MYPT1 was performed in the laboratory of Dr. D. Fulton (Augusta University, Augusta, GA) according to published protocols (Qian *et al*., 2009).

### Ethical approval of the study protocol

The study protocol was approved by the Animal Care and Use Committee of Augusta University (Augusta, GA). The care and treatment of animals was undertaken according to guidelines set by the National Institutes of Health (Bethesda, MD).

### Cell Culture

Human pulmonary artery endothelial cells (HPAEC) obtained from Lonza Group Ltd. (Walkersville, MD) were propagated in culture medium EGM-2-MV (Lonza) supplemented with 5% (v/v) fetal bovine serum (FBS; HyClone, Waltham, MA) and used at passages 3–7. All cells were maintained at 37 °C in a humidified atmosphere of 5% CO_2_.

### Small interfering RNA-mediated depletion

HPAEC were transfected with 50 nM of Ezrin, Radixin, Moesin, CS1β, or MYPT1 small interfering RNA (siRNA) (GE Dharmacon, Lafayette, CO) using SiPort (Thermo Fisher Scientific Ambion, Grand Island, NY) transfection reagent according to the manufacturer’s instructions. Non-targeting siRNA was used as a negative control (Santa Cruz Biotechnology, Santa Cruz, CA).

### Western blot analysis

Immunoblotting was performed as we have described previously^18^,^60^. Membranes were probed with antibodies against MYPT1, phospho-ERM, di-phospho-MLC, CS1β, Ezrin, Radixin, and Moesin. Re-probing with anti-β-actin or GAPDH was used to normalize protein loading.

### Measurements of transendothelial electrical resistance of EC monolayer

Transendothelial electrical resistance (TER) was measured in response to the EC barrier disruptive agent LPS using an electrical cell substrate impedance sensing system (ECIS; Applied Biophysics, Troy, NY) as we have previously described^18^,^60^. HPAEC transfected with siRNA specific to Moesin, Ezrin or Radixin were plated on gold microelectrodes. TER was measured 72 h later.

### Evans Blue Dye (EBD) albumin extravasation into mouse lung

Adult male mice were anaesthetized using 150 mg/kg ketamine and acepromazine. LPS solution (1 mg/kg body weight) or 0.9% sterile saline was introduced intratracheally via a 20-gauge catheter. Animals were allowed to recover for 18 hours. Bronchoalveolar lavage fluid (BALF) and lungs were collected and stored at −80 °C. Evans blue-labeled albumin dye (EBD) was injected into the tail vein 120 minutes before the termination of the experiment in order to assess vascular leak.

### *In vivo* DNA delivery into mouse lung endothelium with JetPEI

*In vivo* gene delivery was performed as we have previously described^27^. Briefly, wild type or D/N CS1β, C/A MYPT1, or phospho-null Moesin mutant in pcDNA 3.1 mammalian expression plasmids (40 μg) or control empty plasmid pcDNA 3.1 (40 μg) were incubated with glucose and jetPEI reagents, as per manufactures instruction for 15–30 minutes. Then, the cDNA/jetPEI complexes were injected into 7–8 week old male mice via the tail vein^27^. At 72 hours post-injection, LPS (1 mg/kg) or sterile saline (0.9%) solution was instilled intratracheally (i.t.) via a 20-gauge catheter. Animals were allowed to recover for 18 hours. After the experiment, lungs were perfused with normal saline through the right ventricle, weighted and snap frozen in liquid nitrogen for further Western blot experiments. Bronchoalveolar lavage fluid (BALF) and lungs were collected and stored at −80 °C. EBD was measured in order to assess vascular leak.

### Lung Histology

Lungs were perfused with PBS and immersed in 4% paraformaldehyde overnight. Sections (5 μm) were cut from paraffin blocks and mounted on slides. Slides were deparafinized in xylene, and run through graded alcohol to distilled water. Slides were pretreated with Target Retrieval Solution (Dako, Carpinteria, CA) using a vegetable steamer and then were rinsed with distilled water. Lung sections were further used for myeloperoxidase staining.

### Measurement of Myeloperoxidase (MPO) Activity

MPO assay kit was used for the staining as we have previously described^27^. Briefly, endogenous peroxidases were quenched with 0.3% H_2_O_2_ for 5 minutes followed by distilled water for 2 minutes, and then washed with 1× PBS for 5 minutes. Slides were treated with Target Retrieval Solution, then incubated in Power Block Solution, rinsed in distilled water, placed in 1× PBS for 5 minutes then incubated with anti-myeloperoxidase (MPO) antibody for 30 minutes at room temperature. The slides were rinsed twice in PBS then incubated with a secondary peroxidase-labeled polymer conjugated to goat anti-rabbit IgG for 30 minutes, and then finally rinsed again in PBS. Bound antibody was detected with diaminobenzidine (DAB + substrate kit). Hematoxylin was used as counter-stain.

### Statistics

Values are expressed as mean ± SEM of at least 3 independent experiments. Analysis of variance (ANOVA) or Student’s t test was used, as appropriate. A *P* value of <0.05 was considered statistically significant.

## Additional Information

**How to cite this article**: Kovacs-Kasa, A. *et al*. The protective role of MLCP-mediated ERM dephosphorylation in endotoxin-induced lung injury *in vitro* and *in vivo* *Sci. Rep.* **6**, 39018; doi: 10.1038/srep39018 (2016).

**Publisher's note:** Springer Nature remains neutral with regard to jurisdictional claims in published maps and institutional affiliations.
